# CRISPR/Cas9‐mediated genome editing: From basic research to translational medicine

**DOI:** 10.1111/jcmm.14916

**Published:** 2020-02-25

**Authors:** Filipe V. Jacinto, Wolfgang Link, Bibiana I. Ferreira

**Affiliations:** ^1^ Centre for Biomedical Research (CBMR) Faro Portugal; ^2^ Departamento de Medicina e Ciências Biomedicas (DCBM) Universidade do Algarve Faro Portugal; ^3^ Algarve Biomedical Center (ABC) Faro Portugal; ^4^ Instituto de Investigaciones Biomédicas “Alberto Sols” (CSIC‐UAM) Madrid Spain

**Keywords:** CRISPR, gene therapy, genome editing, translational medicine

## Abstract

The recent development of the CRISPR/Cas9 system as an efficient and accessible programmable genome‐editing tool has revolutionized basic science research. CRISPR/Cas9 system‐based technologies have armed researchers with new powerful tools to unveil the impact of genetics on disease development by enabling the creation of precise cellular and animal models of human diseases. The therapeutic potential of these technologies is tremendous, particularly in gene therapy, in which a patient‐specific mutation is genetically corrected in order to treat human diseases that are untreatable with conventional therapies. However, the translation of CRISPR/Cas9 into the clinics will be challenging, since we still need to improve the efficiency, specificity and delivery of this technology. In this review, we focus on several in vitro, in vivo and ex vivo applications of the CRISPR/Cas9 system in human disease‐focused research, explore the potential of this technology in translational medicine and discuss some of the major challenges for its future use in patients.

## 1 INTRODUCTION

Genome editing is a type of genetic engineering where DNA is manipulated at the single‐base level. It is revolutionizing the biomedical research field and holds promise to treat or prevent many human genetic disorders. The perfect genome‐editing tool has to be able to alter a genomic sequence efficiently, showing high DNA sequence specificity, and minimal off‐target effects.^1^ Genome‐editing strategies were first developed in yeast 2, 3 and then in mammalian cells,^4^, ^5^ in which small portions of the genome were substituted with exogenous donor DNA sequences via the endogenous homologous recombination repair pathway. In the late 80s, this same natural homologous recombination‐based approach was used in mouse embryonic stem cells to generate mice with a specific genotype.^6^ Since then, this technique has enabled the study of human diseases in mouse and other animal models and contributed considerably in the process of drug discovery and development.

Nevertheless, this approach has several limitations, such as its low editing efficiency and unwanted genome‐editing events where the donor DNA template is more frequently inserted into the genome randomly than at the desired location.^7^ To overcome these limitations, several groups have developed tools that allowed the introduction of site‐specific double‐stranded breaks (DSBs) into a genomic locus of interest using ‘meganucleases’. This refers to endonucleases with an extremely rare recognition site that recognizes and cleaves specific DNA sequences in order to stimulate homology‐directed repair (HDR) mechanism.^8^, ^9^, ^10^, ^11^ This approach requires that a DNA donor template with ends homologous to the break site is delivered and used by the polymerase to copy information along the break site.^9^, ^10^ However, besides HDR, non‐homologous end joining (NHEJ) also occurs at the sites of DSBs.^11^ NHEJ is able to unify the two ends of the break by introducing a random nucleotide insertion or deletion (indels). While NHEJ repair mechanism is exceptionally successful in obtaining functional gene knockouts, the generation of indels emerges as an undesired side effect.^12^ Therefore, the generation of site‐specific DSBs that specifically trigger HDR and simultaneously blunt NHEJ activity is still a current challenge in the field.

Alternatively, site‐directed zinc finger nucleases (ZFs)^13^, ^14^, ^15^ and transcription‐activator‐like effector nucleases (TALENs)^16^, ^17^ are two approaches that use the principles of DNA‐protein recognition. Both ZFs and TALENs are fusion proteins made up of an engineered DNA binding domain fused to a non‐specific nuclease domain from the FokI restriction enzyme. Unlike DNA‐binding proteins, ZF and TALEN amino acid sequences can be designed to cleave virtually any target sequence in the genome with high specificity.^17^, ^18^, ^19^, ^20^, ^21^, ^22^ However, the routine use of these editing tools in the laboratory has been impaired by difficulties in protein design, synthesis and validation.^23^

The development of the CRISPR/Cas9 system has proven to be a major scientific breakthrough and made gene editing more accessible. Distinct from the protein‐guided DNA cleavage used by TALENs and ZFs, CRISPR/Cas9 depends on a small RNA to introduce a site‐specific DSB.^24^, ^25^, ^26^ The requirements of the endonuclease Cas9 to match a DNA target sequence are elegant and simple: It only requires a 20‐nucleotide ‘guideRNA’ (sgRNA) that base pairs with the target DNA and the presence of a DNA ‘protospacer‐adjacent motif’ (PAM), a short DNA sequence adjacent to the complementary region that varies according to the bacterial species of the Cas9 protein being used.^23^, ^24^, ^25^, ^26^, ^27^, ^28^, ^29^ This two‐pronged system in which the sgRNA guides the Cas9 nuclease to target any DNA sequence of interest has replaced the laborious protein design procedure associated with ZFs and TALENs.^1^, ^24^, ^25^, ^26^

The simplicity of CRISPR/Cas9 technology coupled with a unique DNA cleaving mechanism, the ability to target multiple regions, and the existence of different type II CRISPR‐Cas system variants, has enabled notable progresses using this cost‐effective and user‐friendly technology to precise and efficiently modify the genomic DNA of a wide collection of cells and organisms.^23^ Although the CRISPR/Cas9 system has been widely adopted as the preferred genetic editing tool for most researches worldwide, the use of this technology in pre‐clinical and clinical setting is now bursting with new and exciting studies. In this review, we summarize some of the recent disease‐focused studies that have applied the CRISPR/Cas9 system and explore the advantages of this technology as well as discuss the major obstacles involved in translating it to the clinic.

## 2 CRISPR/CAS9: HISTORY AND MECHANISM

In 1987, Ishino et al.^30^ noticed in *Escherichia coli,* the presence of a cluster of repetitive DNA sequences separated by variable spacer regions. Later, Mojica *et al* identified identical type of repeated sequences in numerous bacteria and archaea and named them Clustered Regularly Interspaced Palindromic Repeats or CRISPR.^31^ Interestingly, the biggest breakthrough came in 2005 when the same group realized that these spacer sequences were from unknown origin.^32^, ^33^, ^34^ Together with the observation that many CRISPR‐associated (Cas) genes encode proteins with putative nuclease and helicase domains, it was postulated that CRISPR may constitute an adaptive immunity system 33, 34, 35, 36 by using RNAs as memory signatures of previous infections.^37^ In 2007, Barrangou et al.^38^, using a well‐characterized phage‐sensitive *S thermophilus* strain and two bacteriophages, showed experimentally that CRISPR confers adaptive immunity. In 2008, CRISPR RNAs (crRNAs) were shown to serve as guides in a complex with Cas proteins to promote phage resistance.^39^ The same year, Marraffini and Sontheimer recognized that CRISPR/Cas system was essentially a programmable restriction enzyme targeting DNA.^40^ Interestingly, their paper was the first to explicitly predict that CRISPR might be repurposed for genome editing in heterologous systems. In recent years, work from different groups has been crucial to identify the different components that constitute the recombinant CRISPR/Cas9 system and immense work has been done to demonstrate its functionality in mammalian cells.^1^, ^23^, ^25^, ^27^, ^41^, ^42^

CRISPR mechanisms are very diverse but can be mainly classified into two distinct classes, class 1 and class 2, depending on the organization of the effector protein complex. Class 1 comprehend three different types I, III and IV that are further subdivided into 15 subtypes. Distinct from class 1, that is characterized by the presence of a multi‐protein effector complex, class 2 is defined by a single‐protein effector module. This class is divided into types II, V and VI.^43^ The other CRISPR systems have been extensively reviewed elsewhere.^44^, ^45^ In CRISPR type II, DNA from viruses or plasmids of previous infections is cut into small pieces and integrated into a CRISPR locus amongst short repetitive sequences (30‐40 bp) separated by equally short spacer sequences. The loci are transcribed, and precursor CRISPR RNAs (pre‐crRNAs) are then processed to generate small crRNAs. The pre‐crRNA processing relies on a trans‐activating CRISPR RNA (tracrRNA) that has sequence complementarity to the CRISPR repeat sequence. Upon crRNA:tracrRNA base pairing, which is stabilized by Cas9, endogenous RNAse III cleaves the precursor RNA (pre‐crRNA) into mature crRNAs. The latter are used as guide sequences that will lead Cas nucleases to target and cleave invading DNA based on sequence complementarity. Cleavage of the target sequence, also known as a protospacer, triggers a host immune response by destroying the invader's genome.^23^, ^24^, ^25^, ^26^, ^27^, ^29^, ^46^ The characteristic that makes the type II CRISPR mechanism unique compared to other CRISPR systems is the fact that only one Cas protein (Cas9) is required for gene silencing.^23^, ^27^ During the destruction of target DNA, the two nuclease domains of Cas9, the HNH and RuvC‐like nuclease domains, cleave both DNA strands matching the 20‐nucleotide target sequence resulting in the formation of double‐stranded breaks (DSBs).^25^, ^47^ The HNH domain and the RuvC domains cleave the complementary strand and non‐complementary strand, respectively.^47^ The Cas9 double‐stranded endonuclease activity also requires that a short‐conserved sequence (2‐5 nucleotides), known as protospacer adjacent motif (PAM), is present immediately downstream of the 3´ crRNA. DNA is cleaved three base pairs upstream of the PAM sequence in the complementary DNA strand. In fact, the activity of Cas9 is impaired in the absence of a PAM sequence even if there is complete complementarity by the Cas9‐RNA.^48^ It is important to note that the Cas9 can cleave the non‐complementary DNA strand and generate DSB within 3 bp to 8 bp upstream of the PAM.^25^ This can be of relevance when aiming to perform precise gene editing in a therapeutic setting.

**Figure 1:**
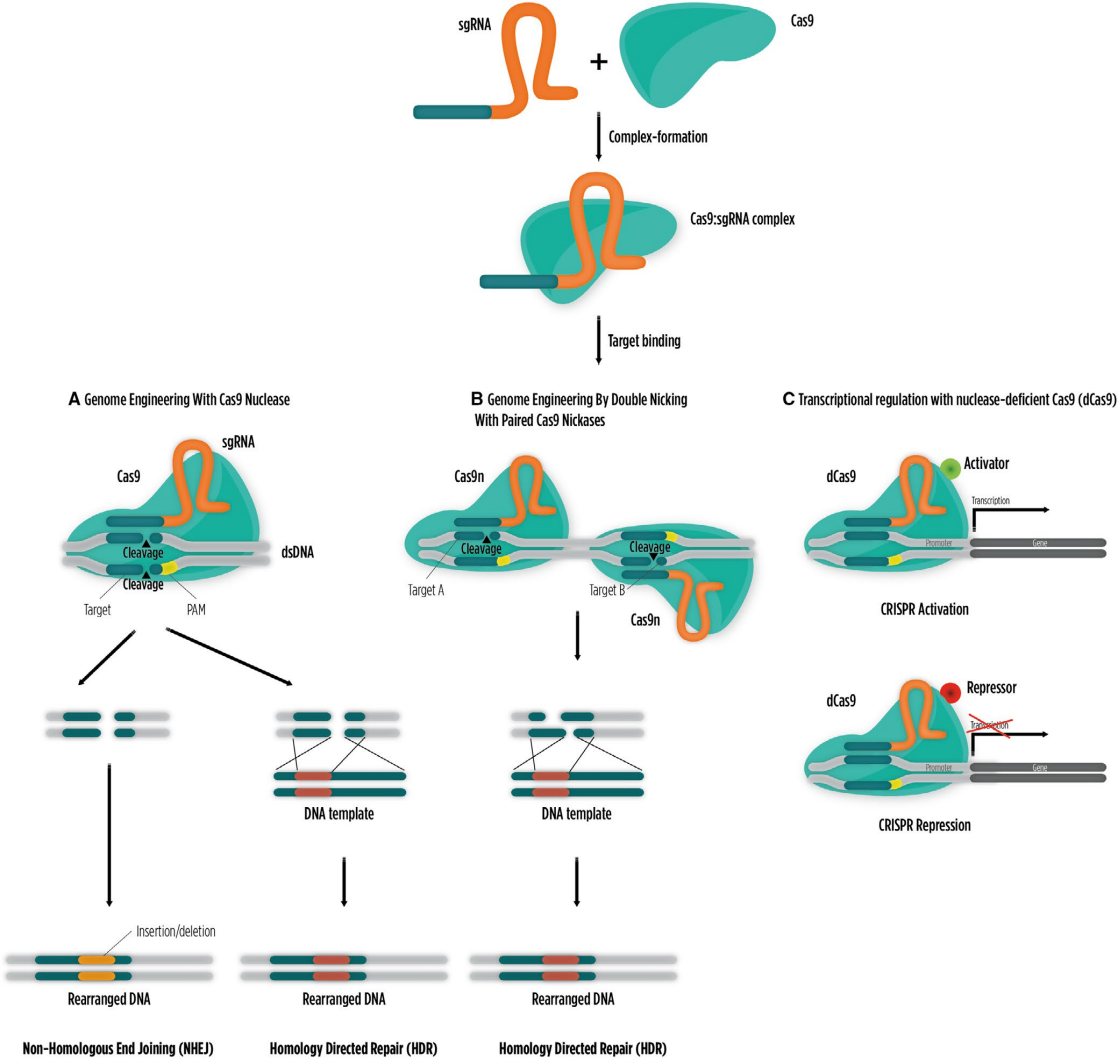
CRISPR/Cas9 genome‐editing tools in mammalian cells. (A) Double‐stranded DNA breaks (DSBs) are generated by CRISPR/Cas9 system, which triggers endogenous DNA repair mechanisms resulting in genetic manipulation. Non‐homologous end joining (NHEJ) is an error‐prone mechanism that is able to disrupt the target gene through the formation of insertions/deletions (indels). Alternatively, homology‐directed repair (HDR) could be activated in the presence of a properly designed DNA repair template to alter a DNA sequence at a specific locus. (B) Mutated Cas9 with only nickase activity (Cas9n) makes a site‐specific single‐stranded nick and does not activate NHEJ. Double‐stranded breaks only occur upon delivery of two sgRNAs that can be later repaired by HDR or NHEJ. (C) Nuclease‐deficient Cas9 (dCas9) can be fused to different effector domains, which allow for the activation or repression of particular target genes in their native context without creating DSBs

The natural occurring type II CRISPR mechanism is a simple three‐component system (Cas9 along with the crRNA and tracRNA) that showed promising potential to be adapted for genome editing. A major milestone came in 2012 when Doudna and Charpentier laboratories developed a simplified two‐component CRISPR/Cas9 system by combining tracRNA and crRNA into a single guide RNA (sgRNA).^25^ This combined version is shown to be as effective as Cas9 programmed with separate tracRNA and crRNA in guiding targeted gene alterations (Figure 1).^25^ The CRISPR/Cas9 system is the most simple, effective and versatile system to date, requiring only the design of a customized sgRNA to generate DSBs at almost any DNA target site. For this reason, this editing technology has quickly widespread within the scientific community to manipulate the genome of numerous cell types and organisms ranging from mice and monkeys to primary human T cells, organoid cultures and stem cells, as well as plants, bacteria and fungi.^49^

## 3 CRISPR/CAS9: AN EFFICIENT TOOL FOR GENOME EDITING IN MAMMALIAN CELLS

### 3.1 In vitro applications

#### 3.1.1 The first studies: a proof of concept

In January 2013, three independent studies have shown that CRISPR/Cas9 mechanism could be repurposed to generate DSBs in DNA. By tweaking this naturally occurring mechanism, researchers were able to perform mammalian genome editing using DNA repair systems, including the NHEJ and the less‐frequent template HDR.^25^, ^42^ NHEJ is the preferred pathway to generate gene knockouts by inducing indels within a coding exon, which might ultimately lead to frameshift mutations and premature stop codons. Alternatively, HDR is used to introduce or alter a specific sequence by using properly designed repair templates (Figure 1A).^25^, ^42^ Cong et al.^42^ developed a more precise variant of the CRISPR/Cas9 system by generating a mutant form that only has nickase activity, known as Cas9D10A or Cas9n. Cas9D10A cuts DNA to generate single‐stranded breaks and does not activate NHEJ. Instead, the HDR repair pathway is activated in the presence of a homologous repair template resulting in reduced indel mutations (Figure 1B). Since 2013, numerous studies have demonstrated the efficacy of this technology by successfully editing the genome of a wide range of cells and organisms.^49^

#### 3.1.2 Application of the CRISPR/Cas9 system in cancer biology

The cancer genetics field is one of the research areas in which CRISPR is having a significant impact. With CRISPR, it is now possible to quickly induce loss‐of‐function (LOF) and gain‐of‐function (GOF) mutations in tumour suppressor genes, oncogenes or other relevant players of the malignant transformation process.^50^ For example, a study by Matano et al. have demonstrated how CRISPR could be used to improve our understanding of human colorectal cancer (CRC) development and progression by introducing serial LOF and GOF mutations frequently associated with CRC in untransformed human intestinal organoids. Surprisingly, the authors found that they could not entirely recapitulate the tumorigenic and metastatic characteristics of this human disease, suggesting that additional genetic and/or epigenetic events are required for the invasive behaviour of CRC.^51^ The CRISPR/Cas9 system is also an invaluable tool to introduce chromosomal translocations that mimic those described in cancers such as lung cancer, acute myeloid leukaemia or Ewing sarcoma.^52^, ^53^, ^54^, ^55^ Human cancer cell lines harbouring these translocations can be obtained by triggering two distant DSBs at defined positions. In addition, the ability to use CRISPR/Cas9 in large‐scale functional screenings offers the opportunity to identify essential genes in various cancer cell lines, uncover genes that are involved in the response to small‐molecule inhibitors or confer resistance to multiple compounds, dissect the relative importance of viral host factors and study combinatorial vulnerabilities.^56^, ^57^, ^58^, ^59^ Most of the CRISPR/Cas9 screens use pooled lentiviral libraries to deliver sgRNAs into the cells. To guarantee high confidence on hit identification, the majority of screens include 3 to 10 sgRNAs per gene.^60^ Remarkable advances using CRISPR/Cas9 technology have been made in cancer immunotherapy. Patient autologous T cells can be genetically edited in vitro to express chimeric antigen receptors (CARs) that specifically recognize and kill tumour cells. These genetically engineered T cells expressing tumour‐targeting receptors have shown therapeutic potential in clinical trials for the treatment of various leukaemias and lymphomas and may eventually be successful in treating solid tumours.^61^ CD19, an antigen expressed by normal B cells and related malignancies, was one of the first targets for CAR T cell–mediated immunotherapy. Currently, different clinical trials are evaluating CAR T therapies targeting antigens in solid tumours, such as Her2/neu, Mesothelin cMet, GD2, interleukin‐13 receptor alpha 2 (IL13Rα2), CEA and EGFR.^60^, ^62^

#### 3.1.3 Application of the CRISPR/Cas9 system in patient‐derived primary and induced pluripotent stem cells

Since the discovery by Yamanaka and colleagues that somatic cells could be reprogrammed into a pluripotent state, human induced pluripotent stem cells (iPSCs) have held great promise in several disease models, regenerative medicine, drug discovery and development.^63^, ^64^ Because CRISPR has shown to be highly efficient at genome editing in iPSCs when compared to alternative systems like TALENs or ZFs, this technology has been commonly used to generate iPSC‐based models of human disease.^65^, ^66^

There are different approaches to generate isogenic disease models in iPSCs using the CRISPR/Cas9 system. For example, it is possible to generate Cas9‐mediated iPSC knockout cell lines via NHEJ that could be used to determine whether a given human mutation is indeed directly responsible for causing the disease or to simply study gene function.^67^, ^68^, ^69^, ^70^ As an alternative approach, specific disease‐related mutations could be introduced into iPSCs using the CRISPR/Cas9 system and HDR‐mediated genome editing to generate in vitro models of human disease.^67^, ^71^ A study by Wang et al.^72^ have demonstrated how CRISPR could be used to help researchers around the world to decipher the underlying cause of human genetic diseases. In this study, the authors shed new light on the pathophysiology underlying the cardiomyopathy of Barth syndrome (BTHS), a mitochondrial disorder caused by a mutation on the tafazzin (TAZ) gene, by combining tissue engineering with patient‐derived and genetically engineered iPSCs.^72^ Furthermore, the authors were able to assess the effect of potential therapies for Barth syndrome using these BTHS iPSC‐derived cardiomyocytes. This pioneering study lays groundwork to develop ‘patient‐to‐patient’ treatment strategies.^72^ Finally, as iPSCs have the capacity to differentiate into any cell type, the generation of genetically engineered iPSCs allows the proper study of human genetic variations in a broad array of tissues in cell culture.^1^

One of the most exciting CRISPR/Cas9 applications with relevance to human health is gene therapy, in which a patient‐specific mutation or mutations are genetically manipulated in order to provide a definitive cure.^1^ Different groups have used in their studies, the CRISPR/Cas9 system to correct human genetic mutations in patient‐derived primary cells, including Fanconi anaemia,^73^ Duchenne muscular dystrophy (DMD),^74^ haemophilia,^75^ cystic fibrosis^76^ and beta thalassaemia.^77^ Additionally, primary immune cells have been edited to knockout the CCR5 or CXCR4 receptor genes using CRISPR/Cas9, resulting in cells resistant to HIV infection.^1^, ^78^, ^79^, ^80^ Together, all these studies highlight the impact that this technology might have in the forthcoming future for the treatment of human genetic disorders.

#### 3.1.4 Application of the CRISPR/Cas9 system in transcriptional regulation

Besides enabling the edition of mammalian genomes, researchers have now the possibility of regulating gene expression and altering epigenetic states. Alterations to the CRISPR/Cas9 system facilitated this new feature without introducing DSBs,^81^ thereby avoiding undesired permanent mutations in target genomic loci. By fusing the viral transcriptional activation domain VP64 to a nuclease‐deficient Cas9 (dCas9), it has been possible to induce the expression of a wide range of genes within their native chromosomal context.^82^, ^83^ This Cas9 version is referred to as CRISPR/Cas9 activation or CRISPRa. However, in the majority of the cases the dCas9‐VP64 system required multiple sgRNAs complementary to the target sequence to achieve strong gene activation.^82^, ^84^ A strategy to boost gene expression levels was to couple several transcriptional activation domains to the dCas9/sgRNA complex (eg tripartite activator system [dCas9‐VPR], synergistic activation mediator [SAM] or dCas9‐SunTag).^85^, ^86^, ^87^ These second‐generation dCas9‐activator fusions proved to exhibit robust transcriptional activation in wide panel of mammalian cell types (Figure 1C).^88^ Furthermore, CRISPRa can be used in genetic screens to unveil molecular targets of novel compounds or to study drug resistance mechanisms in cancer cells. Yang et al.^89^ used a genome‐scale CRISPRa screen and identified *Sall1* as a gene that contributes to reprogramming mouse embryonic fibroblasts into induced pluripotent stem cells.

Conversely, dCas9 has also been utilized in genome‐wide experiments for targeted gene transcriptional repression.^1^, ^23^ Commonly known as CRISPR interference (CRISPRi), this strategy relies on the fact that dCas9 shows high affinity to target DNA and therefore can be repurposed as a transcriptional repressor by blocking transcriptional elongation, RNA polymerase binding and recruitment of transcription repressors.^81^ Moreover, dCas9 can also be fused to the Kruppel‐associated box (KRAB) transcriptional repressor for efficient target gene silencing (Figure 1C).^82^, ^90^, ^91^

Overall, these dCas9 versions that allow for the activation (CRISPRa) or repression (CRISPRi) of target genes are powerful tools that can be used for functional genomic studies under different physiological and developmental conditions without creating DSBs. More recently, an in vivo study on a type I diabetes mouse model repurposed Cas9 to epigenetically induce gene activation and observed a significant improvement on disease phenotypes such as acute kidney injury and muscular dystrophy.^92^ This study further supports that a Cas9‐mediated epigenetic remodelling of target loci could potentially be used as a powerful therapeutic tool to treat several human diseases.

### 3.2 In vivo applications

#### 3.2.1 Application of the CRISPR/Cas9 system in the rapid generation of animal models

CRISPR/Cas9 technology brought a lot of excitement within the scientific community since it revolutionized how fast researchers are able to make a genetically modified animal models.^60^ Previously, the generation of a mouse model was a time‐consuming process that comprehended several laborious steps. Initially, an embryonic stem cell had to be edited to introduce the desired mutation and then injected into the mouse blastocyst. Finally, the offspring had to be screened for germline transmission.^93^ This process was inefficient, labour‐intensive and expensive, which has slowed the generation of genetically engineered animal models. In 2013, the CRISPR/Cas9 system was adapted as an efficient gene‐targeting technology to generate mice carrying mutations in multiple genes in a single editing step by zygote injection.^94^ A few months later, the same group used the CRISPR/Cas9 system to develop a one‐step knock‐in procedure to generate mice carrying reporter and conditional alleles.^95^ Since then, several studies have shown that injecting CRISPR/Cas9 components (Cas9 messenger RNA or protein; sgRNA; HDR template) into a zygote can lead to efficient gene knockout at multiple loci in several animal species, including mice,^96^, ^97^ rat,^97^, ^98^ rabbits^99^ and monkeys,^100^ bypassing targeting in embryonic stem cells. Moreover, the microinjection of zygotes with CRISPR/Cas9 enables researchers to generate additional mutations in pre‐existing animal models of diseases without the need for embryonic stem cell derivation or complex genetic crosses.^60^ Finally, being that CRISPR/Cas9 is a novel genome‐engineering technology that facilitates multiplexed gene targeting, multiple genes can be targeted simultaneously. Therefore, it is easy to obtain mice with multiple gene knockout without the need for crossing single knockout strains.^60^ This is of great interest when the goal is to generate animal models for complex diseases such as cancer. It is important to bear in mind that the majority of the published studies have been performed in murine cancer models that only harbour a low number of mutated genes or alleles.^101^, ^102^ Therefore, the CRISPR/Cas9 system provides an alternative to study cancer in models that resemble the genetic heterogeneity of human cancer genomes. It facilitates the generation of genetically engineered mouse models that harbour mutations in multiple genes involved in cancer progression and also allows the induction of chromosomal translocations or other chromosomal rearrangements, characteristic of many human cancers.^50^ Altogether, CRISPR/Cas9 promises to revolutionize the generation of genetically modified animal models of disease for translational applications by reducing the cost and the time that is necessary to generate in vivo targeted models.

#### 3.2.2 Application of the CRISPR/Cas9 system in animal models for the treatment of human diseases

CRISPR/Cas9 gene‐editing technology has dramatically changed the way we can model and treat human disease in vivo. In order to test the capability of the CRISPR/Cas9 system to correct disease‐causing mutations, in vivo genetically modified animals can be used. In 2013, Wu et al. reported that a mouse model with a dominant cataract‐causing mutation in the Crygc gene could be corrected by co‐injecting Cas9 and a sgRNA targeting the mutant allele into zygotes. CRISPR/Cas9 system was able to correct the mutant allele via HDR pathway using an exogenous oligonucleotide or the endogenous WT allele as template and showing very little off‐target events.^103^ This was one of the first studies that utilized the CRISPR/Cas9 system to efficiently correct a genetic disease. This approach was also used in another study that use the mdx mice, a model of Duchenne muscular dystrophy. It is a rare disorder that is inherited in an X‐linked recessive pattern caused by mutations in the gene that encodes for dystrophin, a protein essential for muscle fibre integrity. DMD is characterized by rapid and progressive muscle weakness and a shortened lifespan, and there is no known cure. Mouse zygotes were injected with Cas9 nuclease, sgRNA and a donor template capable of correcting the *Dmd* gene mutation. This experiment resulted in genetically mosaic progeny ranging 2%–100% gene correction and varying degrees of muscle phenotypic rescue.^104^

In 2014, another study demonstrated that using the mouse model of hereditary tyrosinemia type 1 (HT1), CRISPR/Cas9 system could be used to successfully correct a mutation in post‐natal animals.^105^ They used the Fah59815B mouse model that harbours a homozygous G to A point mutation in the fumarylacetoacetate hydrolase (Fah) gene. This modification induces cytotoxic metabolite accumulation and hepatocyte cell death resulting in severe liver damage. The authors performed hydrodynamic tail vein injection to deliver Cas9 nuclease and a specific sgRNA, along with a single‐stranded DNA (ssDNA) oligo, used as a donor template harbouring the wild‐type G nucleotide for HDR repair, directly into the mouse liver.^105^ Deep sequencing analysis of these animals detected correction of the Fah allele mutation which resulted in protein stabilization, leading to significantly less liver damage. It is important to note that not all hepatocytes have been targeted and corrected. Nonetheless, successfully edited cells were able to survive, expand and repopulate the liver. Hence, disorders where positive selection of edited cells takes place are good candidates for successful gene‐editing therapy.

Since then, other studies have shown that CRISPR/Cas9 gene‐editing technology could be used for the in vivo treatment of other genetic disorders in adult mice. For example, three independent groups have used adeno‐associated virus (AAV) to deliver CRISPR/Cas9 machinery to the mdx mouse model of Duchenne muscular dystrophy with the goal of restoring dystrophin expression in skeletal and cardiac muscle cells.^106^, ^107^, ^108^ This CRISPR/Cas9 method mediated by AAV was able to rescue muscle structure and enhance muscle function in these mice.

Collectively, these in vivo studies set groundbreaking work in the development of new therapies for genetic diseases. For the most part, human genetic disorders are uncurable and this is reflected on patient's poor life quality and shortened life expectancy. The significant progress made in the CRISPR/Cas9 technology enables the development of promising treatments and highlights the potential of this gene therapy approach to cure human genetic diseases.^1^

#### 3.2.3 Application of the CRISPR/Cas9 system in cardiovascular disease

Cardiovascular diseases (CVDs) are the number one cause of death worldwide according to the World Health Organization. The advances in CRISPR/Cas9 technology have shown to greatly impact the development of new in vivo tools that allow a better understanding of mechanisms underlying CVDs. More importantly, the expansion of CRISPR/Cas9 techniques accelerated the development of novel therapies capable of treating CVDs.^109^ Accordingly, the number of published studies reporting on CRISPR/Cas9 applications to the field of CVD has increased significantly during the recent years. As discussed in the previous section, DMD has proven to be a model disease where CRISPR/Cas9 technology clearly has been shown to be successful. Likewise, other heritable cardiomyopathies are promising candidates for genome‐editing therapies. Ma et al. 110 described for the first time the use of CRISPR/Cas9 to effectively correct a disease‐causing mutation in the MYCBPC3 gene in human embryos. MYCBPC3 mutations are the pre‐eminent cause of hypertrophic cardiomyopathy (HCM), a common inherited disorder that results in the abnormal thickening of the left ventricular wall.^111^ The authors found that co‐injecting Cas9 with sperm into M‐phase oocytes resulted in 72.4% of embryos showing a homozygous wild‐type genotype.^110^ Another group was able to extend survival and improve cardiac function after ablating the PLN gene using CRISPR/Cas9 in a transgenic mice overexpressing a model of severe heart failure.^112^

The availability of viable and efficient delivery methods represents one of the biggest challenges of translating the CRISPR/Cas9 into the clinic, as it will be discussed in more detail in the following section. Finn et al. 113 reported the development of a lipid nanoparticle system capable of genetically editing the transthyretin (*Ttr*) gene in the mouse liver with a single administration. The authors combined the nanoparticle system with CRISPR/Cas9 components to target the *Ttr* gene and observed a significant reduction in serum protein levels that persisted for at least 12 months. It will be interesting to test whether this approach will be effective and durable in other disease models other than cardiac amyloidosis.

#### 3.2.4 Application of the CRISPR/Cas9 system in ex vivo gene therapy

The success of ex vivo gene therapy relies on the establishment of optimized protocols for culturing patient‐derived primary cells that after genome editing can be transplanted back into the patient. The hematopoietic system is an excellent target for this approach, because target cells can be easily withdrawn from the patient peripheral blood and can be re‐injected after editing and expansion.^60^ Clinical trials using ZFs as a tool for ex vivo gene therapy are being conducted on patients with several blood disorders, including severe‐combined immunodeficiency, Fanconi anaemia, Wiskott‐Aldrich syndrome and sickle‐cell anaemia.^114^, ^115^ Recently, a clinical trial has shown that gene editing can be used in humans to test and treat HIV safe and effectively.^116^ In this study, ZFs were used to disrupt the C‐C motif chemokine receptor 5 (CCR5), the major co‐receptor used by HIV strains to infect T cells. The infusion of autologous T cells genetically edited at the CCR5 locus resulted in the partial induction of acquired genetic resistance to HIV infection.^116^ This approach is now being tested in phase 1/2 clinical trials. However, genetically manipulated T cells do not self‐renew and so this treatment could only be effective for a specific period of time. The disruption of CCR5 in human self‐renewing hematopoietic stem cells (HSC) as shown by Holt et al. 117 using ZFs could potentially solve this limitation. A more recent study used the CRISPR/Cas9 gene‐editing technology to target the CCR5 gene in human CD34+ hematopoietic stem and progenitor stem cells (HSPCs). HSPCs that were successfully edited via CRISPR/Cas9 technology maintained multi‐lineage potential.^118^ Another important example of ex vivo CRISPR/Cas9 application is the CAR T cell–mediated immunotherapy discussed in more detail in the ‘cancer biology’ section above.

The precise selection of genetically modified cells harbouring the correct edited allele without undesirable off‐target mutations represents one of the most important aspects of ex vivo gene therapy. As the process of selection is very efficient, and only selected cells will be transferred back into the patient, the accuracy of CRISPR/Cas9 is less critical in ex vivo than in in vivo gene therapy.^119^ However, one of the major downsides of ex vivo approaches is that additional genomic alterations can occur during the required cell expansion step in culture. This is of pivotal importance, as the cells used for the gene‐editing step are normally stem/progenitor cells susceptible to accumulate mutations and copy number variations during reprogramming and expansion. Accordingly, it will be important to develop assays to measure the integrity and normal functioning of genetically modified stem/progenitor cells before advancing a therapy to the clinics. Nevertheless, despite these challenges, the CRISPR/Cas9 genome‐editing tool shows enormous potential for bringing ex vivo gene therapy into the clinic in a near future.

#### 3.2.5 CRISPR/Cas9‐mediated genome‐editing applications in translational medicine: the challenges and the future

CRISPR/Cas9 system is undoubtedly the most revolutionary technology in medicine over the past decades. Since researchers were able to demonstrate that CRISPR/Cas9 could be repurposed as an editing tool, this technology has developed with enormous scientific, medical and industry impact. In this review, we have covered several examples of how CRISPR/Cas9 technology had direct or indirect impact in the clinic. Despite recent and important advances, several issues have to be addressed in order to bring CRISPR/Cas9 genome‐editing tools into the clinic. One of the major obstacles for the translation of CRISPR/Cas9 into clinically useful tools is the possible off‐target effects. Several studies using a variety of approaches, including computational predictions, in vitro and in vivo high‐throughput profiling and whole‐genome sequencing methods, have reported significant rates of off‐target effects associated with CRISPR/Cas9. It is important to note that unwanted effects of CRISPR/Cas9 such as off‐target editing and off‐target binding might result in malignant transformation and other unforeseeable consequences.^120^, ^121^, ^122^, ^123^, ^124^, ^125^, ^126^, ^127^, ^128^, ^129^

The development of methods that minimize off‐target effects of CRISPR/Cas9 approaches has been a major focus of research. One of these strategies requires that two separate Cas9 binding events simultaneously occur at the same locus in order to execute cleavage of DNA. The inactivation of either of the two catalytic residues within Cas9 converts the enzyme into a nickase (Cas9n) which cleaves or ‘nicks’ a single DNA strand instead of a double strand.^1^, ^130^ By generating two distinct sgRNAs, DSBs only take place with simultaneous binding events because separate Cas9ns nick opposite DNA strands. As the probability of two off‐target sites being adjacent in the genome is low, this strategy increases the stringency significantly.^130^ Alternatively, the dimerizing FokI nuclease domain which is part of the genome‐editing tools ZFS and TALLENs can be fused to a nuclease‐deficient dCas9 and thereby induce DSBs exclusively upon paired binding.^131^, ^132^, ^133^ A recent strategy to limit off‐target events is based on sgRNA or protein engineering increasing specificity.^134^, ^135^, ^136^, ^137^ Enhanced nuclease Cas9 (eSpCas9) and SpCas9‐HF1 are two examples of Cas9 variations that have been modified for that effect.^135^, ^137^ A different strategy that reduces off‐target events and enhances Cas9 specificity relies on reducing Cas9 lifetime or activity in cells after time modifying the target locus. For example, the use of tightly titrated amounts of Cas9‐sgRNA ribonucleotide protein complexes (RNPs), known to be rapidly degraded, can improve the ratio between on‐target and off‐target genome editing in mammalian cells, dramatically.^1^, ^41^, ^138^ Recently, a paper was published describing an improved Cas9 version named xCas9 capable of recognizing a wide range of PAM sequences including GAA, GAT and NG.^139^ This novel variant overcomes an important limitation associated with the CRISPR/Cas9 system, the requirement of a PAM sequence at the target site. In 2016, Abudayyeh et al. 140 characterized a novel type of nuclease, the Cas13a, and showed its RNA‐guided ribonuclease function. A single CRISPR RNA guides this nuclease that can cleave single‐stranded RNA targets within bacteria and mammalian cells.^140^, ^141^ The class 2 CRISPR‐Cas family is very diverse comprising three distinct types (II, V and VI). Until now, there are four known subtypes that belong to type VI, Cas13a, Cas13b, Cas13c and Cas13d.^28^, ^140^, ^141^, ^142^ Cas13d shows robust knock‐down across many endogenous transcripts, and since is one of the smallest Cas proteins, it can fit within the packaging limits of a AAV for in vivo delivery.^142^ More interestingly, Cas13 nuclease has non‐specific RNAse activity highlighting its potential as a diagnostics tool.^143^, ^144^ Besides cutting the target DNA or RNA, this family of nucleases is able to cleave surrounding single‐stranded RNAs (ssRNA). This unique property facilitated the development of diagnostic kits by using ssRNA reporter molecules that fluoresce upon Cas nuclease activity towards a specific disease‐guided RNA.

Another major obstacle for the clinical translation of CRISPR/Cas9 is the limited efficiency of HDR‐mediated gene correction. Factors known to determine this efficiency include cell type, cell state and competition with the NHEJ. As many treatments of human genetic diseases are based on HDR‐mediated gene correction, in which a template sequence is delivered to replace the mutated version, major progress in the efficiency of HDR is necessary. Several efforts have been made to increase HDR efficacy, including the rational design of single‐stranded DNA donors.^145^ Importantly, the design of the sgRNA is also critical to ensure complementarity to the target sequence and minimize off‐target cleavage. It has been described that mismatches at the proximal 5’ region, relative to the PAM sequence, are better tolerated than those at the 3’ region.^146^ Therefore, design of sgRNA should avoid mismatches further away from the PAM as it increases the probability of off‐target events. Another strategy is inhibiting the NHEJ pathway^147^, ^148^ or increasing the similarity of the donor template and the double‐stranded break sites.^149^ ‘Base editing’ is a recent method of genome engineering that facilitates direct, irreversible conversion of a specific target DNA base into another through RNA‐programmed mechanism, without a dsDNA backbone cleavage or the need of a donor template. ‘Base editing’ could represent an alternative to HDR‐mediated gene correction.^150^ Fusion of dCas9 to a cytidine deaminase enzyme, that acts on single‐stranded DNA, allows C to U conversion within a window as small as approximately five nucleotides. The fused enzyme is capable of efficiently correcting a range of disease‐relevant point mutations.^150^ Another group has also developed an adenine base editor that mediates conversion of AT to GC in genomic DNA using a tRNA adenosine deaminase fused with Cas9.^151^

Besides its low efficiency, HDR has been considered to be mainly limited to applications in dividing cells.^152^ This fact represents an important setback for its broad use in the treatment of human genetic diseases, making it challenging to apply the technique to post‐mitotic cells. However, more recently it has been shown that adeno‐associated virus (AAV)–mediated delivery of donor template in combination with DNA cleavage by CRISPR/Cas9 allows for precise genome editing through HDR in post‐mitotic neurons in mouse brain.^153^ Another challenge imposed by the need of correcting specific mutations refers to the mutational variability amongst patients with the same disease. This becomes a big hurdle to overcome when there is the need of designing patient‐tailored sgRNAs and DNA donor templates. In particular, customizing CRISPR/Cas9 gene therapy drugs represents a major challenge for effectively scaling production in the future.^154^

Virtually, all macromolecular therapies have to solve issues of delivery that often limit their efficacy.^155^ Efficient in vivo gene therapy using CRISPR/Cas9 will depend on the efficient and tissue‐specific delivery of its components. The majority of in vivo studies report the delivery of therapeutic CRISPR/Cas9 components through viral vectors, especially AAV.^156^, ^157^, ^158^ AAV vectors engineered for gene therapy seem particularly promising because they can infect both dividing and non‐dividing cells, they do not integrate into the host genome, and they fail to induce a significant host immune response and efficiently transduce a broad range of cell types.^159^ However, AAVs have a limited packaging capacity for foreign DNA of ≃4.5 kb.^160^ Consequently, it is generally not possible packaging all the CRISPR/Cas9 components, including the *Streptococcus pyogenes* Cas9 (spCas9) gene(4.2 Kb), the sgRNA, the donor template as well as associated promoters and regulatory sequences into AAV.^1^ Strikingly, a recent study used a significantly smaller Cas9 gene (3.2 Kb) from *Staphylococcus aureus* (saCas9) allowing for the integration of a Cas9 together with a sgRNA into a single AAV.^129^ Alternatively, the genes coding for SpCas9 and its sgRNA can be packaged into separate AAV vectors as demonstrated for in vivo CRISPR/Cas9‐mediated genome editing in mouse brains^161^ and livers.^162^

Host immune responses induced by the delivery of bacterial Cas9 proteins or gene therapy vectors represent another challenge for the translation of CRISPR/Cas9 approaches into the clinic. More recently, a mouse model for non‐alcoholic steatohepatitis (NASH), a frequent liver disease in humans characterized by excessive fat build‐up in the liver, was generated using spCas9 to delete *Pten*, a tumour suppressor gene involved in NASH and a repressor of the PI3/AKT pathway. Surprisingly, this study describes the production of Cas9‐specific antibodies and the secretion of IL‐2 from splenocytes that had been engineered with Cas9 system targeting the *Pten* locus.^163^ In this study, the Cas9 was delivered by adenoviral vectors, known to trigger an immune response and might have enhanced that outcome.^164^ A promising way to avoid the immunogenicity of viral vectors is the use of non‐viral vectors including nanoparticle‐ and lipid‐based vectors.^165^, ^166^ A possible strategy to limit the immunogenicity of Cas9 peptides is humanizing the Cas9 protein.^154^ Accordingly, finding methods that reduce the immunogenicity of the in vivo CRISPR/Cas9‐mediated gene editing will be an important focus of upcoming researches.

### 3.3 Ethical concerns

The burst of CRISPR/Cas9 applications also highlighted the potential of this system and the ethical concerns associated with the possible creation of permanent and inheritable changes in the human genome. Having in mind the ethical implications, legal action was taken to delay germline genome editing. The first report using CRISPR in human embryos was back in 2015 where Liang et al. 167 performed experiments in discarded embryos with an extra pair of chromosomes. Nonetheless, gene correction efficiency was very low and the successful ones showed genetic mosaicism with a low percentage of cells being accurately edited. Another group reported that they had successfully edited three out of six viable human embryos.^168^ They have used immature oocytes that had to go through in vitro maturation. More recently, Ma et al. 110 utilized CRISPR in human diploid zygotes to correct a mutation causative of hypertrophic cardiomyopathy, a congenital heart disease, claiming high‐efficiency and few side effects. The group of Dr Huang at ShangaiTech University used a new CRISPR method, the recently developed base editing (discussed in the previous section) to correct a single base in the *FBN1* gene involved in the Marfan syndrome, a rare autosomal dominant disorder in heterozygous embryos.^169^ The embryos were obtained by injecting sperm from a patient with Marfan syndrome into a mature oocyte. The authors showed that 89% of the embryos were efficiently edited, and more importantly, no off‐target and indels were detected. It is evident that we are on an accelerated pace towards using CRISPR genomic engineering as a biomedical therapy. But is also urgent that discussions about ethical guidelines within international multidisciplinary groups take place to regulate and minimize the potential risks associated with this powerful tool.

## 4 CONCLUSIONS

The CRISPR/Cas9 RNA‐guided DNA endonuclease system is a versatile technology that has rapidly transformed genome editing and basic science research. The development of improved CRISPR/Cas9 tools with high degree of DNA specificity, increased selectivity and low level of by‐products made this technology accessible to researchers worldwide to study human diseases. For example, it is now feasible to generate in vivo animal models of specific diseases in a few weeks. It is now possible to envision the treatment of genetic diseases in the near future using this technology. In fact, several clinical trials using CRISPR/Cas9 approach to treat human genetic diseases are underway (NCT03872479 or NCT03399448). However, we still need to improve the efficiency, specificity and delivery of this technology for its broader application in the clinics. A major concern that accompanies the use of CRISPR/Cas9 in the clinical setting relates to the potential risk of misusing this technology. The development of ethical and regulatory guidelines is critical to ensure that the benefits outweigh and minimize the risks. The discovery of CRISPR/Cas9 technology and its application in the clinics is a true example of the importance of bridging basic research and translational medicine. Once the CRISPR/Cas9 mechanism was unveiled, the possibilities of medical exploitation were enormous and will definitely change the way we will treat genetic disorders in the future.

## CONFLICT OF INTEREST

The authors declare that they have no conflict of interest.

## AUTHOR CONTRIBUTION

FV.J performed research and wrote the manuscript. WL wrote the manuscript. BI.F conceptualized the study, edited, wrote and submitted the manuscript.

## References

[1] Komor AC , Badran AH , Liu DR . CRISPR‐based technologies for the manipulation of eukaryotic genomes. Cell. 2017;169:559. 10.1016/j.cell.2017.04.005 28431253

[2] Hinnen A , Hicks JB , Fink GR . Transformation of yeast. Proc. Natl. Acad. Sci. USA. 1978;1992:337‐341. 10.1073/pnas.75.4.1929 PMC392455 347451

[3] Scherer S , Davis RW . Replacement of chromosome segments with altered DNA sequences constructed in vitro. Proc Natl Acad Sci USA. 1979;76:4951‐4955. 388424 10.1073/pnas.76.10.4951 PMC413056

[4] Thomas KR , Capecchi MR . Targeting of genes to specific sites in the mammalian genome. Cold Spring Harb Symp Quant Biol. 1986;51(Pt 2):1101‐1113. 3472755 10.1101/sqb.1986.051.01.128

[5] Smithies O , Gregg RG , Boggs SS , et al. Insertion of DNA sequences into the human chromosomal beta‐globin locus by homologous recombination. Nature. 1985;317:230‐234. 2995814 10.1038/317230a0

[6] Capecchi MR . Altering the genome by homologous recombination. Science. 1989;244:1288‐1292. 2660260 10.1126/science.2660260

[7] Lin FL , Sperle K , Sternberg N . Recombination in mouse L cells between DNA introduced into cells and homologous chromosomal sequences. Proc Natl Acad Sci USA. 1985;82:1391‐1395. 3856266 10.1073/pnas.82.5.1391 PMC397267

[8] Rudin N , Sugarman E , Haber JE . Genetic and physical analysis of double‐strand break repair and recombination in Saccharomyces cerevisiae. Genetics. 1989;122:519‐534. 2668114 10.1093/genetics/122.3.519 PMC1203726

[9] Haber JE . Partners and pathways repairing a double‐strand break. Trends Genet. 2000;16:259‐264. 10827453 10.1016/s0168-9525(00)02022-9

[10] Jasin M , Rothstein R . Repair of strand breaks by homologous recombination. Cold Spring Harb Perspect Biol. 2013;5:a012740. 24097900 10.1101/cshperspect.a012740 PMC3809576

[11] Eid A , Mahfouz MM . Genome editing: the road of CRISPR/Cas9 from bench to clinic. Exp Mol Med. 2016;48:e265‐e275. 27741224 10.1038/emm.2016.111 PMC5099421

[12] Chapman JR , Taylor MRG , Boulton SJ . Playing the end game: DNA double‐strand break repair pathway choice. Mol Cell. 2012;47:497‐510. 22920291 10.1016/j.molcel.2012.07.029

[13] Bibikova M , Beumer K , Trautman JK , et al. Enhancing gene targeting with designed zinc finger nucleases. Science. 2003;300:764‐774. 12730594 10.1126/science.1079512

[14] Porteus MH , Baltimore D . Chimeric nucleases stimulate gene targeting in human cells. Science. 2003;300:763‐773. 12730593 10.1126/science.1078395

[15] Urnov FD , Rebar EJ , Holmes MC , et al. Genome editing with engineered zinc finger nucleases. Nat Rev Genet. 2010;11:636‐646. 20717154 10.1038/nrg2842

[16] Li T , Huang S , Zhao X , et al. Modularly assembled designer TAL effector nucleases for targeted gene knockout and gene replacement in eukaryotes. Nucl Acids Res. 2011;39:6315‐6325. 21459844 10.1093/nar/gkr188 PMC3152341

[17] Joung JK , Sander JD . TALENs: a widely applicable technology for targeted genome editing. Nat Rev Mol Cell Biol. 2013;14:49‐55. 23169466 10.1038/nrm3486 PMC3547402

[18] Carroll D . Progress and prospects: zinc‐finger nucleases as gene therapy agents. Gene Ther. 2008;15:1463‐1468. 18784746 10.1038/gt.2008.145 PMC2747807

[19] Boch J , Scholze H , Schornack S , et al. Breaking the code of DNA binding specificity of TAL‐type III effectors. Science. 2009;326:1509‐1512. 19933107 10.1126/science.1178811

[20] Miller JC , Tan S , Qiao G , et al. A TALE nuclease architecture for efficient genome editing. Nat. Biotechnol. 2011;29:143‐148. 21179091 10.1038/nbt.1755

[21] Moscou MJ , Bogdanove AJ . A simple cipher governs DNA recognition by TAL effectors. Science. 2009;326:1501‐1511. 19933106 10.1126/science.1178817

[22] Zhang F , Cong L , Lodato S , et al. Efficient construction of sequence‐specific TAL effectors for modulating mammalian transcription. Nat Biotechnol. 2011;29:149‐153. 21248753 10.1038/nbt.1775 PMC3084533

[23] Doudna JA , Charpentier E . Genome editing. The new frontier of genome engineering with CRISPR‐Cas9. Science. 2014;346:1258096‐1258106. 25430774 10.1126/science.1258096

[24] Gasiunas G , Barrangou R , Horvath P , et al. Cas9‐crRNA ribonucleoprotein complex mediates specific DNA cleavage for adaptive immunity in bacteria. Proc Natl Acad Sci USA. 2012;109:E2579‐E2586. 22949671 10.1073/pnas.1208507109 PMC3465414

[25] Jinek M , Chylinski K , Fonfara I , et al. A programmable dual‐RNA‐guided DNA endonuclease in adaptive bacterial immunity. Science. 2012;337:816‐821. 22745249 10.1126/science.1225829 PMC6286148

[26] Garneau JE , Dupuis M‐È , Villion M , et al. The CRISPR/Cas bacterial immune system cleaves bacteriophage and plasmid DNA. Nature. 2010;468:67‐71. 21048762 10.1038/nature09523

[27] Barrangou R , Horvath P . A decade of discovery: CRISPR functions and applications. Nat Microbiol. 2017;2:17092. 28581505 10.1038/nmicrobiol.2017.92

[28] Hsu PD , Lander ES , Zhang F . Development and applications of CRISPR‐Cas9 for genome engineering. Cell. 2014;157:1262‐1278. 24906146 10.1016/j.cell.2014.05.010 PMC4343198

[29] Sapranauskas R , Gasiunas G , Fremaux C , et al. The Streptococcus thermophilus CRISPR/Cas system provides immunity in Escherichia coli. Nucleic Acids Res. 2011;39:9275‐9282. 21813460 10.1093/nar/gkr606 PMC3241640

[30] Ishino Y , Shinagawa H , Makino K , et al. Nucleotide sequence of the iap gene, responsible for alkaline phosphatase isozyme conversion in *Escherichia coli*, and identification of the gene product. J Bacteriol. 1987;169:5429‐5433. 3316184 10.1128/jb.169.12.5429-5433.1987 PMC213968

[31] Mojica FJ , Díez‐Villaseñor C , Soria E , et al. Biological significance of a family of regularly spaced repeats in the genomes of Archaea, Bacteria and mitochondria. Mol Microbiol. 2000;36:244‐246. 10760181 10.1046/j.1365-2958.2000.01838.x

[32] Mojica FJM , Díez‐Villaseñor C , García‐Martínez J , et al. Intervening sequences of regularly spaced prokaryotic repeats derive from foreign genetic elements. J. Mol. Evol. 2005;60:174‐182. 15791728 10.1007/s00239-004-0046-3

[33] Pourcel C , Salvignol G , Vergnaud G . CRISPR elements in Yersinia pestis acquire new repeats by preferential uptake of bacteriophage DNA, and provide additional tools for evolutionary studies. Microbiology. 2005;151:653‐663. 15758212 10.1099/mic.0.27437-0

[34] Bolotin A , Quinquis B , Sorokin A , et al. Clustered regularly interspaced short palindrome repeats (CRISPRs) have spacers of extrachromosomal origin. Microbiology. 2005;151:2551‐2561. 16079334 10.1099/mic.0.28048-0

[35] Jansen R , Embden JDAV , Gaastra W , et al. Identification of genes that are associated with DNA repeats in prokaryotes. Mol Microbiol. 2002;43:1565‐1575. 11952905 10.1046/j.1365-2958.2002.02839.x

[36] Haft DH , Selengut J , Mongodin EF , et al. A guild of 45 CRISPR‐associated (Cas) protein families and multiple CRISPR/Cas subtypes exist in prokaryotic genomes. PLoS Comput Biol. 2005;1:e60. 16292354 10.1371/journal.pcbi.0010060 PMC1282333

[37] Makarova KS , Grishin NV , Shabalina SA , et al. A putative RNA‐interference‐based immune system in prokaryotes: computational analysis of the predicted enzymatic machinery, functional analogies with eukaryotic RNAi, and hypothetical mechanisms of action. Biol Direct. 2006;1:7. 16545108 10.1186/1745-6150-1-7 PMC1462988

[38] Barrangou R , Fremaux C , Deveau H , et al. CRISPR provides acquired resistance against viruses in prokaryotes. Science. 2007;315:1709‐1712. 17379808 10.1126/science.1138140

[39] Brouns SJJ , Jore MM , Lundgren M , et al. Small CRISPR RNAs guide antiviral defense in prokaryotes. Science. 2008;321:960‐964. 18703739 10.1126/science.1159689 PMC5898235

[40] Marraffini LA , Sontheimer EJ . CRISPR interference limits horizontal gene transfer in staphylococci by targeting DNA. Science. 2008;322:1843‐1845. 19095942 10.1126/science.1165771 PMC2695655

[41] Cho SW , Kim S , Kim JM , et al. Targeted genome engineering in human cells with the Cas9 RNA‐guided endonuclease. Nat Biotechnol. 2013;31:230‐232. 23360966 10.1038/nbt.2507

[42] Cong L , Ran FA , Cox D , et al. Multiplex genome engineering using CRISPR/Cas systems. Science. 2013;339:819‐823. 23287718 10.1126/science.1231143 PMC3795411

[43] Koonin EV , Makarova KS , Zhang F . Diversity, classification and evolution of CRISPR‐Cas systems. Curr Opin Microbiol. 2017;37:67‐78. 28605718 10.1016/j.mib.2017.05.008 PMC5776717

[44] Wright AV , Nuñez JK , Doudna JA . Biology and applications of CRISPR systems: Harnessing nature's toolbox for genome engineering. Cell. 2016;164:29‐44. 26771484 10.1016/j.cell.2015.12.035

[45] Makarova KS , Wolf YI , Alkhnbashi OS , et al. An updated evolutionary classification of CRISPR‐Cas systems. Nat Rev Microbiol. 2015;13:722‐736. 26411297 10.1038/nrmicro3569 PMC5426118

[46] Deltcheva E , Chylinski K , Sharma CM , et al. CRISPR RNA maturation by trans‐encoded small RNA and host factor RNase III. Nature. 2011;471:602‐607. 21455174 10.1038/nature09886 PMC3070239

[47] Nishimasu H , Ran FA , Hsu PD , et al. Crystal structure of Cas9 in complex with guide RNA and target DNA. Cell. 2014;156:935‐949. 24529477 10.1016/j.cell.2014.02.001 PMC4139937

[48] Sternberg SH , Redding S , Jinek M , et al. DNA interrogation by the CRISPR RNA‐guided endonuclease Cas9. Nature. 2014;507:62‐67. 24476820 10.1038/nature13011 PMC4106473

[49] Sternberg SH , Doudna JA . Expanding the Biologist's Toolkit with CRISPR‐Cas9. Mol Cell. 2015;58:568‐574. 26000842 10.1016/j.molcel.2015.02.032

[50] Sánchez‐Rivera FJ , Jacks T . Applications of the CRISPR‐Cas9 system in cancer biology. Nat Rev Cancer. 2015;15:387‐395. 26040603 10.1038/nrc3950 PMC4530801

[51] Matano M , Date S , Shimokawa M , et al. Modeling colorectal cancer using CRISPR‐Cas9‐mediated engineering of human intestinal organoids. Nat Med. 2015;21:256‐262. 25706875 10.1038/nm.3802

[52] Chen C , Liu Y , Rappaport AR , et al. MLL3 is a haploinsufficient 7q tumor suppressor in acute myeloid leukemia. Cancer Cell. 2014;25:652‐665. 24794707 10.1016/j.ccr.2014.03.016 PMC4206212

[53] Choi PS , Meyerson M . Targeted genomic rearrangements using CRISPR/Cas technology. Nat Commun. 2014;5:3728. 24759083 10.1038/ncomms4728 PMC4170920

[54] Torres R , Martin MC , Garcia A , et al. Engineering human tumour‐associated chromosomal translocations with the RNA‐guided CRISPR‐Cas9 system. Nat Commun. 2014;5:3964. 24888982 10.1038/ncomms4964

[55] Ghezraoui H , Piganeau M , Renouf B , et al. Chromosomal translocations in human cells are generated by canonical nonhomologous end‐joining. Mol Cell. 2014;55:829‐842. 25201414 10.1016/j.molcel.2014.08.002 PMC4398060

[56] Wang T , Birsoy K , Hughes NW , et al. Identification and characterization of essential genes in the human genome. Science. 2015;350:1096‐1101. 26472758 10.1126/science.aac7041 PMC4662922

[57] Shalem O , Sanjana NE , Zhang F . High‐throughput functional genomics using CRISPR‐Cas9. Nat Rev Genet. 2015;16:299‐311. 25854182 10.1038/nrg3899 PMC4503232

[58] Hart T , Chandrashekhar M , Aregger M , et al. High‐resolution CRISPR screens reveal fitness genes and genotype‐specific cancer liabilities. Cell. 2015;163:1515‐1526. 26627737 10.1016/j.cell.2015.11.015

[59] Koike‐Yusa H , Li Y , Tan E‐P , et al. Genome‐wide recessive genetic screening in mammalian cells with a lentiviral CRISPR‐guide RNA library. Nat Biotechnol. 2014;32:267‐273. 24535568 10.1038/nbt.2800

[60] Fellmann C , Gowen BG , Lin P‐C , et al. Cornerstones of CRISPR‐Cas in drug discovery and therapy. Nat Rev Drug Discov. 2017;16:89‐100. 28008168 10.1038/nrd.2016.238 PMC5459481

[61] Maus MV , Grupp SA , Porter DL , et al. Antibody‐modified T cells: CARs take the front seat for hematologic malignancies. Blood. 2014;123:2625‐2635. 24578504 10.1182/blood-2013-11-492231 PMC3999751

[62] Ren J , Zhao Y . Advancing chimeric antigen receptor T cell therapy with CRISPR/Cas9. Protein Cell. 2017;8:634‐643. 28434148 10.1007/s13238-017-0410-x PMC5563282

[63] Takahashi K , Yamanaka S . Induction of pluripotent stem cells from mouse embryonic and adult fibroblast cultures by defined factors. Cell. 2006;126:663‐676. 16904174 10.1016/j.cell.2006.07.024

[64] Yu J , Vodyanik MA , Smuga‐Otto K , et al. Induced pluripotent stem cell lines derived from human somatic cells. Science. 2007;318:1917‐1920. 18029452 10.1126/science.1151526

[65] Ding Q , Regan SN , Xia Y , et al. Enhanced efficiency of human pluripotent stem cell genome editing through replacing TALENs with CRISPRs. Cell Stem Cell. 2013;12:393‐394. 23561441 10.1016/j.stem.2013.03.006 PMC3925309

[66] Hockemeyer D , Jaenisch R . Induced pluripotent stem cells meet genome editing. Cell Stem Cell. 2016;18:573‐586. 27152442 10.1016/j.stem.2016.04.013 PMC4871596

[67] Smith C , Abalde‐Atristain L , He C , et al. Efficient and allele‐specific genome editing of disease loci in human iPSCs. Mol Ther. 2015;23:570‐577. 25418680 10.1038/mt.2014.226 PMC4351458

[68] Liao J , Karnik R , Gu H , et al. Targeted disruption of DNMT1, DNMT3A and DNMT3B in human embryonic stem cells. Nat Genet. 2015;47:469‐478. 25822089 10.1038/ng.3258 PMC4414868

[69] González F , Zhu Z , Shi Z‐D , et al. An iCRISPR platform for rapid, multiplexable, and inducible genome editing in human pluripotent stem cells. Cell Stem Cell. 2014;15:215‐226. 24931489 10.1016/j.stem.2014.05.018 PMC4127112

[70] Chen Y , Cao J , Xiong M , et al. Engineering Human Stem Cell Lines with Inducible Gene Knockout using CRISPR/Cas9. Cell Stem Cell. 2015;17:233‐244. 26145478 10.1016/j.stem.2015.06.001 PMC4530040

[71] Hou Z , Zhang Y , Propson NE , et al. Efficient genome engineering in human pluripotent stem cells using Cas9 from Neisseria meningitidis. Proc Natl Acad Sci USA. 2013;110:15644‐15649. 23940360 10.1073/pnas.1313587110 PMC3785731

[72] Wang G , McCain ML , Yang L , et al. Modeling the mitochondrial cardiomyopathy of Barth syndrome with induced pluripotent stem cell and heart‐on‐chip technologies. Nat Med. 2014;20:616‐623. 24813252 10.1038/nm.3545 PMC4172922

[73] Osborn MJ , Gabriel R , Webber BR , et al. Fanconi anemia gene editing by the CRISPR/Cas9 system. Hum Gene Ther. 2015;26:114‐126. 25545896 10.1089/hum.2014.111 PMC4326027

[74] Ousterout DG , Kabadi AM , Thakore PI , et al. Multiplex CRISPR/Cas9‐based genome editing for correction of dystrophin mutations that cause Duchenne muscular dystrophy. Nat Commun. 2015;6:6244. 25692716 10.1038/ncomms7244 PMC4335351

[75] Park C‐Y , Kim DH , Son JS , et al. Functional correction of large factor VIII gene chromosomal inversions in Hemophilia A patient‐derived iPSCs using CRISPR‐Cas9. Cell Stem Cell. 2015;17:213‐220. 26212079 10.1016/j.stem.2015.07.001

[76] Schwank G , Koo B‐K , Sasselli V , et al. Functional repair of CFTR by CRISPR/Cas9 in intestinal stem cell organoids of cystic fibrosis patients. Cell Stem Cell. 2013;13:653‐658. 24315439 10.1016/j.stem.2013.11.002

[77] Xie F , Ye L , Chang JC , et al. Seamless gene correction of β‐thalassemia mutations in patient‐specific iPSCs using CRISPR/Cas9 and piggyBac. Genome Res. 2014;24:1526‐1533. 25096406 10.1101/gr.173427.114 PMC4158758

[78] Wang W , Ye C , Liu J , et al. CCR5 gene disruption via lentiviral vectors expressing Cas9 and single guided RNA renders cells resistant to HIV‐1 infection. PLoS One. 2014;9:e115987. 25541967 10.1371/journal.pone.0115987 PMC4277423

[79] Li C , Guan X , Du T , et al. Inhibition of HIV‐1 infection of primary CD4+ T‐cells by gene editing of CCR5 using adenovirus‐delivered CRISPR/Cas9. J Gen Virol. 2015;96:2381‐2393. 25854553 10.1099/vir.0.000139

[80] Schumann K , Lin S , Boyer E , et al. Generation of knock‐in primary human T cells using Cas9 ribonucleoproteins. Proc Natl Acad Sci USA. 2015;112:10437‐10442. 26216948 10.1073/pnas.1512503112 PMC4547290

[81] Qi LS , Larson MH , Gilbert LA , et al. Repurposing CRISPR as an RNA‐guided platform for sequence‐specific control of gene expression. Cell. 2013;152:1173‐1183. 23452860 10.1016/j.cell.2013.02.022 PMC3664290

[82] Gilbert LA , Larson MH , Morsut L , et al. CRISPR‐mediated modular RNA‐guided regulation of transcription in eukaryotes. Cell. 2013;154:442‐451. 23849981 10.1016/j.cell.2013.06.044 PMC3770145

[83] Perez‐Pinera P , Kocak DD , Vockley CM , et al. RNA‐guided gene activation by CRISPR‐Cas9‐based transcription factors. Nat Meth. 2013;10:973‐976. 10.1038/nmeth.2600 PMC3911785 23892895

[84] Kearns NA , Genga RMJ , Enuameh MS , et al. Cas9 effector‐mediated regulation of transcription and differentiation in human pluripotent stem cells. Development. 2014;141:219‐223. 24346702 10.1242/dev.103341 PMC3865759

[85] Chavez A , Scheiman J , Vora S , et al. Highly efficient Cas9‐mediated transcriptional programming. Nat Meth. 2015;12:326‐328. 10.1038/nmeth.3312 PMC4393883 25730490

[86] Konermann S , Brigham MD , Trevino AE , et al. Genome‐scale transcriptional activation by an engineered CRISPR‐Cas9 complex. Nature. 2015;517:583‐588. 25494202 10.1038/nature14136 PMC4420636

[87] Tanenbaum ME , Gilbert LA , Qi LS , et al. A protein‐tagging system for signal amplification in gene expression and fluorescence imaging. Cell. 2014;159:635‐646. 25307933 10.1016/j.cell.2014.09.039 PMC4252608

[88] Chavez A , Tuttle M , Pruitt BW , et al. Comparison of Cas9 activators in multiple species. Nat Meth. 2016;13:563‐567. 10.1038/nmeth.3871 PMC4927356 27214048

[89] Yang J , Rajan SS , Friedrich MJ , et al. Genome‐scale CRISPRa screen identifies novel factors for cellular reprogramming. Stem Cell Rep. 2019;12:757‐771. 10.1016/j.stemcr.2019.02.010 PMC6450436 30905739

[90] Lawhorn IEB , Ferreira JP , Wang CL . Evaluation of sgRNA target sites for CRISPR‐mediated repression of TP53. PLoS One. 2014;9:e113232. 25398078 10.1371/journal.pone.0113232 PMC4232525

[91] Thakore PI , D'Ippolito AM , Song L , et al. Highly specific epigenome editing by CRISPR‐Cas9 repressors for silencing of distal regulatory elements. Nat Meth. 2015;12:1143‐1149. 10.1038/nmeth.3630 PMC4666778 26501517

[92] Liao H‐K , Hatanaka F , Araoka T , et al. In vivo target gene activation via CRISPR/Cas9‐mediated trans‐epigenetic modulation. Cell. 2017;171(1495–1507):e15. 10.1016/j.cell.2017.10.025 PMC5732045 29224783

[93] Capecchi MR . Gene targeting in mice: functional analysis of the mammalian genome for the twenty‐first century. Nat Rev Genet. 2005;6:507‐512. 15931173 10.1038/nrg1619

[94] Wang H , Yang H , Shivalila CS , et al. One‐step generation of mice carrying mutations in multiple genes by CRISPR/Cas‐mediated genome engineering. Cell. 2013;153:910‐918. 23643243 10.1016/j.cell.2013.04.025 PMC3969854

[95] Yang H , Wang H , Shivalila CS , et al. One‐step generation of mice carrying reporter and conditional alleles by CRISPR/Cas‐mediated genome engineering. Cell. 2013;154:1370‐1379. 23992847 10.1016/j.cell.2013.08.022 PMC3961003

[96] Shen B , Zhang J , Wu H , et al. Generation of gene‐modified mice via Cas9/RNA‐mediated gene targeting. Cell Res. 2013;23:720‐723. 23545779 10.1038/cr.2013.46 PMC3641603

[97] Li D , Qiu Z , Shao Y , et al. Heritable gene targeting in the mouse and rat using a CRISPR‐Cas system. Nat Biotechnol. 2013;31:681‐683. 23929336 10.1038/nbt.2661

[98] Li W , Teng F , Li T , et al. Simultaneous generation and germline transmission of multiple gene mutations in rat using CRISPR‐Cas systems. Nat Biotechnol. 2013;31:684‐686. 23929337 10.1038/nbt.2652

[99] Yang D , Xu J , Zhu T , et al. Effective gene targeting in rabbits using RNA‐guided Cas9 nucleases. J Mol Cell Biol. 2014;6:97‐99. 24403564 10.1093/jmcb/mjt047 PMC3983410

[100] Chen Y , Zheng Y , Kang Y , et al. Functional disruption of the dystrophin gene in rhesus monkey using CRISPR/Cas9. Hum Mol Genet. 2015;24:3764‐3774. 25859012 10.1093/hmg/ddv120 PMC5007610

[101] Guerra C , Mijimolle N , Dhawahir A , et al. Tumor induction by an endogenous K‐ras oncogene is highly dependent on cellular context. Cancer Cell. 2003;4:111‐120. 12957286 10.1016/s1535-6108(03)00191-0

[102] Jackson EL , Willis N , Mercer K , et al. Analysis of lung tumor initiation and progression using conditional expression of oncogenic K‐ras. Genes Dev. 2001;15:3243‐3248. 11751630 10.1101/gad.943001 PMC312845

[103] Wu Y , Liang D , Wang Y , et al. Correction of a genetic disease in mouse via use of CRISPR‐Cas9. Cell Stem Cell. 2013;13:659‐662. 24315440 10.1016/j.stem.2013.10.016

[104] Long C , McAnally JR , Shelton JM , et al. Prevention of muscular dystrophy in mice by CRISPR/Cas9‐mediated editing of germline DNA. Science. 2014;345:1184‐1188. 25123483 10.1126/science.1254445 PMC4398027

[105] Yin H , Xue W , Chen S , et al. Genome editing with Cas9 in adult mice corrects a disease mutation and phenotype. Nat Biotechnol. 2014;32:551‐553. 24681508 10.1038/nbt.2884 PMC4157757

[106] Long C , Amoasii L , Mireault AA , et al. Postnatal genome editing partially restores dystrophin expression in a mouse model of muscular dystrophy. Science. 2016;351:400‐403. 26721683 10.1126/science.aad5725 PMC4760628

[107] Nelson CE , Hakim CH , Ousterout DG , et al. In vivo genome editing improves muscle function in a mouse model of Duchenne muscular dystrophy. Science. 2016;351:403‐407. 26721684 10.1126/science.aad5143 PMC4883596

[108] Tabebordbar M , Zhu K , Cheng JKW , et al. In vivo gene editing in dystrophic mouse muscle and muscle stem cells. Science. 2016;351:407‐411. 26721686 10.1126/science.aad5177 PMC4924477

[109] German DM , Mitalipov S , Mishra A , et al. Therapeutic Genome Editing in Cardiovascular Diseases. JACC Basic Transl Sci. 2019;4:122‐131. 30847427 10.1016/j.jacbts.2018.11.004 PMC6390678

[110] Ma H , Marti‐Gutierrez N , Park S‐W , et al. Correction of a pathogenic gene mutation in human embryos. Nature. 2017;548:413‐419. 28783728 10.1038/nature23305

[111] Viswanathan SK , Sanders HK , McNamara JW , et al. Hypertrophic cardiomyopathy clinical phenotype is independent of gene mutation and mutation dosage. PLoS One. 2017;12:e0187948. 29121657 10.1371/journal.pone.0187948 PMC5679632

[112] Kaneko M , Hashikami K , Yamamoto S , et al. Phospholamban Ablation Using CRISPR/Cas9 System Improves Mortality in a Murine Heart Failure Model. PLoS One. 2016;11:e0168486. 27992596 10.1371/journal.pone.0168486 PMC5161475

[113] Finn JD , Smith AR , Patel MC , et al. A single administration of CRISPR/Cas9 lipid nanoparticles achieves robust and persistent in vivo genome editing. Cell Rep. 2018;22:2227‐2235. 29490262 10.1016/j.celrep.2018.02.014

[114] Jo Y‐I , Kim H , Ramakrishna S . Recent developments and clinical studies utilizing engineered zinc finger nuclease technology. Cell Mol Life Sci. 2015;72:3819‐3830. 26089249 10.1007/s00018-015-1956-5 PMC11113831

[115] Hoban MD , Cost GJ , Mendel MC , et al. Correction of the sickle cell disease mutation in human hematopoietic stem/progenitor cells. Blood. 2015;125:2597‐2604. 25733580 10.1182/blood-2014-12-615948 PMC4408287

[116] Tebas P , Stein D , Tang WW , et al. Gene editing of CCR5 in autologous CD4 T cells of persons infected with HIV. N Engl J Med. 2014;370:901‐910. 24597865 10.1056/NEJMoa1300662 PMC4084652

[117] Holt N , Wang J , Kim K , et al. Human hematopoietic stem/progenitor cells modified by zinc‐finger nucleases targeted to CCR5 control HIV‐1 in vivo. Nat Biotechnol. 2010;28:839‐847. 20601939 10.1038/nbt.1663 PMC3080757

[118] Mandal PK , Ferreira LMR , Collins R , et al. Efficient ablation of genes in human hematopoietic stem and effector cells using CRISPR/Cas9. Cell Stem Cell. 2014;15:643‐652. 25517468 10.1016/j.stem.2014.10.004 PMC4269831

[119] Savić N , Schwank G . Advances in therapeutic CRISPR/Cas9 genome editing. Transl Res. 2016;168:15‐21. 26470680 10.1016/j.trsl.2015.09.008

[120] Cencic R , Miura H , Malina A , et al. Protospacer adjacent motif (PAM)‐distal sequences engage CRISPR Cas9 DNA target cleavage. PLoS One. 2014;9:e109213. 25275497 10.1371/journal.pone.0109213 PMC4183563

[121] Kuscu C , Arslan S , Singh R , et al. Genome‐wide analysis reveals characteristics of off‐target sites bound by the Cas9 endonuclease. Nat Biotechnol. 2014;32:677‐683. 24837660 10.1038/nbt.2916

[122] Wu X , Scott DA , Kriz AJ , et al. Genome‐wide binding of the CRISPR endonuclease Cas9 in mammalian cells. Nat Biotechnol. 2014;32:670‐676. 24752079 10.1038/nbt.2889 PMC4145672

[123] O'Geen H , Henry IM , Bhakta MS , et al. A genome‐wide analysis of Cas9 binding specificity using ChIP‐seq and targeted sequence capture. Nucl Acids Res. 2015;43:3389‐3404. 25712100 10.1093/nar/gkv137 PMC4381059

[124] Smith C , Gore A , Yan W , et al. Whole‐genome sequencing analysis reveals high specificity of CRISPR/Cas9 and TALEN‐based genome editing in human iPSCs. Cell Stem Cell. 2014;15:12‐13. 24996165 10.1016/j.stem.2014.06.011 PMC4338993

[125] Veres A , Gosis BS , Ding Q , et al. Low incidence of off‐target mutations in individual CRISPR‐Cas9 and TALEN targeted human stem cell clones detected by whole‐genome sequencing. Cell Stem Cell. 2014;15:27‐30. 24996167 10.1016/j.stem.2014.04.020 PMC4082799

[126] Yang L , Grishin D , Wang G , et al. Targeted and genome‐wide sequencing reveal single nucleotide variations impacting specificity of Cas9 in human stem cells. Nat Commun. 2014;5:5507. 25425480 10.1038/ncomms6507 PMC4352754

[127] Kim D , Bae S , Park J , et al. Digenome‐seq: genome‐wide profiling of CRISPR‐Cas9 off‐target effects in human cells. Nat Meth. 2015;12:237–43–1pfollowing243. 10.1038/nmeth.3284 25664545

[128] Tsai SQ , Zheng Z , Nguyen NT , et al. GUIDE‐seq enables genome‐wide profiling of off‐target cleavage by CRISPR‐Cas nucleases. Nat Biotechnol. 2015;33:187‐197. 25513782 10.1038/nbt.3117 PMC4320685

[129] Ran FA , Cong L , Yan WX , et al. In vivo genome editing using Staphylococcus aureus Cas9. Nature. 2015;520:186‐191. 25830891 10.1038/nature14299 PMC4393360

[130] Ran FA , Hsu PD , Lin C‐Y , et al. Double nicking by RNA‐guided CRISPR Cas9 for enhanced genome editing specificity. Cell. 2013;154:1380‐1389. 23992846 10.1016/j.cell.2013.08.021 PMC3856256

[131] Guilinger JP , Thompson DB , Liu DR . Fusion of catalytically inactive Cas9 to FokI nuclease improves the specificity of genome modification. Nat Biotechnol. 2014;32:577‐582. 24770324 10.1038/nbt.2909 PMC4263420

[132] Tsai SQ , Wyvekens N , Khayter C , et al. Dimeric CRISPR RNA‐guided FokI nucleases for highly specific genome editing. Nat Biotechnol. 2014;32:569‐576. 24770325 10.1038/nbt.2908 PMC4090141

[133] Wyvekens N , Topkar VV , Khayter C , et al. Dimeric CRISPR RNA‐Guided FokI‐dCas9 Nucleases Directed by Truncated gRNAs for Highly Specific Genome Editing. Hum Gene Ther. 2015;26:425‐431. 26068112 10.1089/hum.2015.084 PMC4509490

[134] Fu Y , Sander JD , Reyon D , et al. Improving CRISPR‐Cas nuclease specificity using truncated guide RNAs. Nat Biotechnol. 2014;32:279‐284. 24463574 10.1038/nbt.2808 PMC3988262

[135] Slaymaker IM , Gao L , Zetsche B , et al. Rationally engineered Cas9 nucleases with improved specificity. Science. 2016;351:84‐88. 26628643 10.1126/science.aad5227 PMC4714946

[136] Kleinstiver BP , Prew MS , Tsai SQ , et al. Engineered CRISPR‐Cas9 nucleases with altered PAM specificities. Nature. 2015;523:481‐485. 26098369 10.1038/nature14592 PMC4540238

[137] Kleinstiver BP , Pattanayak V , Prew MS , et al. High‐fidelity CRISPR‐Cas9 nucleases with no detectable genome‐wide off‐target effects. Nature. 2016;529:490‐495. 26735016 10.1038/nature16526 PMC4851738

[138] Lin S , Staahl BT , Alla RK , et al. Enhanced homology‐directed human genome engineering by controlled timing of CRISPR/Cas9 delivery. Elife. 2014;3:e04766. 25497837 10.7554/eLife.04766 PMC4383097

[139] Hu JH , Miller SM , Geurts MH , et al. Evolved Cas9 variants with broad PAM compatibility and high DNA specificity. Nature. 2018;556:57‐63. 29512652 10.1038/nature26155 PMC5951633

[140] Abudayyeh OO , Gootenberg JS , Konermann S , et al. C2c2 is a single‐component programmable RNA‐guided RNA‐targeting CRISPR effector. Science. 2016;353:aaf5573. 27256883 10.1126/science.aaf5573 PMC5127784

[141] Abudayyeh OO , Gootenberg JS , Essletzbichler P , et al. RNA targeting with CRISPR‐Cas13. Nature. 2017;550:280‐284. 28976959 10.1038/nature24049 PMC5706658

[142] Konermann S , Lotfy P , Brideau NJ , et al. Transcriptome engineering with RNA‐targeting type VI‐D CRISPR effectors. Cell. 2018;173: 665‐676.e14. 29551272 10.1016/j.cell.2018.02.033 PMC5910255

[143] Cox DBT , Gootenberg JS , Abudayyeh OO , et al. RNA editing with CRISPR‐Cas13. Science. 2017;358:1019‐1027. 29070703 10.1126/science.aaq0180 PMC5793859

[144] Gootenberg JS , Abudayyeh OO , Lee JW , et al. Nucleic acid detection with CRISPR‐Cas13a/C2c2. Science. 2017;356:438‐442. 28408723 10.1126/science.aam9321 PMC5526198

[145] Richardson CD , Ray GJ , DeWitt MA , et al. Enhancing homology‐directed genome editing by catalytically active and inactive CRISPR‐Cas9 using asymmetric donor DNA. Nat Biotechnol. 2016;34:339‐344. 26789497 10.1038/nbt.3481

[146] Fu Y , Foden JA , Khayter C , et al. High‐frequency off‐target mutagenesis induced by CRISPR‐Cas nucleases in human cells. Nat Biotechnol. 2013;31:822‐826. 23792628 10.1038/nbt.2623 PMC3773023

[147] Chu VT , Weber T , Wefers B , et al. Increasing the efficiency of homology‐directed repair for CRISPR‐Cas9‐induced precise gene editing in mammalian cells. Nat Biotechnol. 2015;33:543‐548. 25803306 10.1038/nbt.3198

[148] Maruyama T , Dougan SK , Truttmann MC , et al. Increasing the efficiency of precise genome editing with CRISPR‐Cas9 by inhibition of nonhomologous end joining. Nat Biotechnol. 2015;33:538‐542. 25798939 10.1038/nbt.3190 PMC4618510

[149] Moynahan ME , Jasin M . Mitotic homologous recombination maintains genomic stability and suppresses tumorigenesis. Nat Rev Mol Cell Biol. 2010;11:196‐207. 20177395 10.1038/nrm2851 PMC3261768

[150] Komor AC , Kim YB , Packer MS , et al. Programmable editing of a target base in genomic DNA without double‐stranded DNA cleavage. Nature. 2016;533:420‐424. 27096365 10.1038/nature17946 PMC4873371

[151] Gaudelli NM , Komor AC , Rees HA , et al. Programmable base editing of A•T to G•C in genomic DNA without DNA cleavage. Nature. 2017;551:464‐471. 29160308 10.1038/nature24644 PMC5726555

[152] Rothkamm K , Krüger I , Thompson LH , et al. Pathways of DNA double‐strand break repair during the mammalian cell cycle. Mol Cell Biol. 2003;23:5706‐5715. 12897142 10.1128/MCB.23.16.5706-5715.2003 PMC166351

[153] Nishiyama J , Mikuni T , Yasuda R . Virus‐mediated genome editing via homology‐directed repair in mitotic and postmitotic cells in mammalian brain. Neuron. 2017;96:755‐765. 29056297 10.1016/j.neuron.2017.10.004 PMC5691606

[154] Dai W‐J , Zhu L‐Y , Yan Z‐Y , et al. CRISPR‐Cas9 for in vivo Gene Therapy: Promise and Hurdles. Mol Ther Nucleic Acids. 2016;5:e349. 28131272 10.1038/mtna.2016.58 PMC5023403

[155] Lino CA , Harper JC , Carney JP , et al. Delivering CRISPR: a review of the challenges and approaches. Drug Deliv. 2018;25:1234‐1257. 29801422 10.1080/10717544.2018.1474964 PMC6058482

[156] Maddalo D , Manchado E , Concepcion CP , et al. In vivo engineering of oncogenic chromosomal rearrangements with the CRISPR/Cas9 system. Nature. 2014;516:423‐427. 25337876 10.1038/nature13902 PMC4270925

[157] Cheng R , Peng J , Yan Y , et al. Efficient gene editing in adult mouse livers via adenoviral delivery of CRISPR/Cas9. FEBS Lett. 2014;588:3954‐3958. 25241167 10.1016/j.febslet.2014.09.008

[158] Ding Q , Strong A , Patel KM , et al. Permanent alteration of PCSK9 with in vivo CRISPR‐Cas9 genome editing. Circ Res. 2014;115:488‐492. 24916110 10.1161/CIRCRESAHA.115.304351 PMC4134749

[159] Wang AY , Peng PD , Ehrhardt A , et al. Comparison of adenoviral and adeno‐associated viral vectors for pancreatic gene delivery in vivo. Hum Gene Ther. 2004;15:405‐413. 15053865 10.1089/104303404322959551

[160] Wu Z , Yang H , Colosi P . Effect of genome size on AAV vector packaging. Mol Ther. 2010;18:80‐86. 19904234 10.1038/mt.2009.255 PMC2839202

[161] Swiech L , Heidenreich M , Banerjee A , et al. In vivo interrogation of gene function in the mammalian brain using CRISPR‐Cas9. Nat Biotechnol. 2015;33:102‐106. 25326897 10.1038/nbt.3055 PMC4492112

[162] Yang Y , Wang L , Bell P , et al. A dual AAV system enables the Cas9‐mediated correction of a metabolic liver disease in newborn mice. Nat Biotechnol. 2016;34:334‐338. 26829317 10.1038/nbt.3469 PMC4786489

[163] Wang D , Mou H , Li S , et al. Adenovirus‐Mediated Somatic Genome Editing of Pten by CRISPR/Cas9 in Mouse Liver in Spite of Cas9‐Specific Immune Responses. Hum Gene Ther. 2015;26:432‐442. 26086867 10.1089/hum.2015.087 PMC4509492

[164] Geutskens SB , van der Eb MM , Plomp AC , et al. Recombinant adenoviral vectors have adjuvant activity and stimulate T cell responses against tumor cells. Gene Ther. 2000;7:1410‐1416. 10981668 10.1038/sj.gt.3301251

[165] Zuris JA , Thompson DB , Shu Y , et al. Cationic lipid‐mediated delivery of proteins enables efficient protein‐based genome editing in vitro and in vivo. Nat Biotechnol. 2015;33:73‐80. 25357182 10.1038/nbt.3081 PMC4289409

[166] Yin H , Song C‐Q , Dorkin JR , et al. Therapeutic genome editing by combined viral and non‐viral delivery of CRISPR system components in vivo. Nat Biotechnol. 2016;34:328‐333. 26829318 10.1038/nbt.3471 PMC5423356

[167] Liang P , Xu Y , Zhang X , et al. CRISPR/Cas9‐mediated gene editing in human tripronuclear zygotes. Protein Cell. 2015;6:363‐372. 25894090 10.1007/s13238-015-0153-5 PMC4417674

[168] Tang L , Zeng Y , Du H , et al. CRISPR/Cas9‐mediated gene editing in human zygotes using Cas9 protein. Mol Genet Genomics. 2017;292:525‐533. 28251317 10.1007/s00438-017-1299-z

[169] Zeng Y , Li J , Li G , et al. Correction of the Marfan Syndrome Pathogenic FBN1 Mutation by Base Editing in Human Cells and Heterozygous Embryos. Mol Ther. 2018;26:2631‐2637. 30166242 10.1016/j.ymthe.2018.08.007 PMC6224777

